# Combining direct current and kilohertz frequency alternating current to mitigate onset activity during electrical nerve block

**DOI:** 10.1088/1741-2552/abebed

**Published:** 2021-03-22

**Authors:** Thomas Eggers, Joseph Kilgore, David Green, Tina Vrabec, Kevin Kilgore, Niloy Bhadra

**Affiliations:** 1 Emory University School of Medicine, Atlanta, GA, United States of America; 2 MetroHealth Medical Center, Cleveland, OH, United States of America; 3 Department of Biomedical Engineering, Case Western Reserve University, Cleveland, OH, United States of America; 4 Louis Stokes Cleveland Department Veterans Affairs Medical Center, Cleveland, OH, United States of America

**Keywords:** electrical nerve block, kilohertz frequency alternating current, direct current nerve block, sciatic nerve, functional electrical stimulation

## Abstract

**Objective.:**

Electrical nerve block offers the ability to immediately and reversibly block peripheral nerve conduction and would have applications in the emerging field of bioelectronics. Two modalities of electrical nerve block have been investigated—kilohertz frequency alternating current (KHFAC) and direct current (DC). KHFAC can be safely delivered with conventional electrodes, but has the disadvantage of having an onset response, which is a period of increased neural activation before block is established and currently limits clinical translation. DC has long been known to block neural conduction without an onset response but creates damaging reactive species. Typical electrodes can safely deliver DC for less than one second, but advances in high capacitance electrodes allow DC delivery up to 10 s without damage. The present work aimed to combine DC and KHFAC into a single waveform, named the combined reduced onset waveform (CROW), which can initiate block without an onset response while also maintaining safe block for long durations. This waveform consists of a short, DC pre-pulse before initiating KHFAC.

**Approach.:**

Simulations of this novel waveform were carried out in the axonal simulation environment NEURON to test feasibility and gain insight into the mechanisms of action. Two sets of acute experiments were then conducted in adult Sprague–Dawley rats to determine the effectiveness of the waveform in mitigating the onset response.

**Main results.:**

The CROW reduced the onset response both *in silico* and *in vivo*. The onset area was reduced by over 90% with the tested parameters in the acute experiments. The amplitude of the DC pulse was shown to be particularly important for effective onset mitigation, requiring amplitudes 6–8 times the DC block threshold.

**Significance.:**

This waveform can reliably reduce the onset response due to KHFAC and could allow for wider clinical implementation of electrical nerve block.

## 1. Introduction

Electrical nerve block is a promising neuromodulation technique which offers the ability to directly down-regulate neural activity. Blocking unwanted or pathogenic neural activity offers the ability to treat an array of diseases and conditions, such as pain [1], spasticity or autonomic conditions such as cardiac arrhythmias [2]. Unlike current methods to block or inhibit neural activity, e.g. pharmaceuticals, electrical nerve block creates near instantaneous block (<1 s) which can also be reversed instantly [3–5]. Electrical nerve block also provides a graded block; fiber selectivity is possible with larger fibers blocked at lower thresholds than smaller fibers [3, 6, 7]. Two modalities of electrical nerve block have been studied in recent years—kilohertz frequency alternating current (KHFAC) and direct current (DC). A detailed review of electric nerve block has been published recently [8].

KHFAC delivers a charge balanced waveform, typically in the form of a sinusoid, and thus can safely deliver charge for hours [9] or possibly indefinitely. The mechanism of action of KHFAC neural block is postulated to be a dynamic steady-state depolarization leading to a reduced population of open sodium channels [10, 11]. KHFAC is already being tested in human clinical trials to treat post-amputation neuroma pain (Neuros Medical) [1], treat obesity [12], and for the mitigation of low back pain. The main disadvantage that currently limits KHFAC from more widespread application is the presence of an onset response [5]. The onset response is a period of neural activation that occurs when KHFAC is first initiated, lasting anywhere from <1 s to >30 s in motor nerves [8]. The onset has two phases, which we refer to as Phase I and Phase II [5]. The Phase I onset lasts for approximately 100 ms, and results in a single summated twitch (when tested in a motor nerve) [13], likely due to synchronous activation of multiple fibers. Phase II follows Phase I and lasts anywhere between one to over 30 s, and can produce low intensity but sustained onset activity, likely due to asynchronous firing of a subset of fibers. Our lab has studied several methods to reduce the onset response [14–17], with varying success.

DC has long been known to create localized nerve block [4]. The mechanism of action in cathodic DC block is through depolarization, producing inactivation of sodium channels [4, 18, 19]. DC was not used for practical applications due to the risk of neural damage. DC can create reactive species which damage underlying neural structures if the charge capacity of the electrode is exceeded [20]. For a typically-sized platinum electrode (2 × 3 mm^2^), this charge capacity is exceeded in ≪1 s [21], making DC block impractical for any clinical application. Recent developments in this field include electrodes with higher charge capacity [22], charge balancing of the DC waveform using the high charge capacity electrodes [23, 24], physical separation of the electrode (and the generated reactants) from the neural tissue [25], or even ‘rectification’ of the current with a novel device [26]. These modifications can greatly extend the ability of electrodes to safely deliver DC. Even with these advances, a small implanted electrode on a nerve can only safely block for <10 s. However, DC has the distinct advantage over KHFAC that a short amplitude ramp can eliminate any onset response when block is initiated [27].

In this work, we combined these two modalities to create an electrical nerve block which can be initiated with little to no onset and be safely maintained for hours. Previous attempts at this design include providing the DC and KHFAC through two separate electrodes [28] and adding a DC offset to a KHFAC waveform [29]. This study uses a novel waveform which combines separate DC and KHFAC phases delivered through a monopolar electrode. DC initiates the block, and is then rapidly switched to KHFAC to maintain the block with a minimal onset response at the transition. Initially the waveform was studied in an axonal computer model. Following this, an *in-vivo* experimental investigation was conducted. The amplitude and duration of the DC portion of the waveform was systematically and randomly varied to determine the optimal values to reduce the onset response. The timing between the DC and KHFAC was also varied to investigate the influence of gap times on the contribution of DC to onset mitigation.

## 2. Methods

### 2.1. Simulations

#### 2.1.1. Simulation environment

Computer simulations were carried out in NEURON (version 7.6.2), a nerve simulation software [30]. All simulations utilized the myelinated mammalian axon model (MRG model), developed by McIntyre *et al* [31] and further extended to include a frequency-dependent membrane capacitance based on Howell *et al* [32]. The nerve membrane model consisted of quantitatively defined equivalent electrical circuits for both the nodes and internode regions of the axon [31, 33]. The node model circuit consisted of a parallel combination of a nonlinear fast sodium conductance, a nonlinear persistent sodium conductance, a nonlinear slow potassium conductance, a linear leakage conductance, and a membrane capacitance. The internode region was modeled as a circuit composed of two layers of linear components. The frequency-dependent membrane capacitance was modeled by adding a series capacitor and resistor in parallel with the other components in all regions [32]. Howell *et al*, have shown that block thresholds (BTs) of sinusoids are reduced with the inclusion of a frequency-dependent membrane capacitance by as much as 11%. The inclusion of this dependence improves upon the previous MRG model which was not originally validated for kilohertz frequency extracellular stimulation. The specific parameters used were based on McIntyre *et al* and are shown in table 1 [31].

#### 2.1.2. Simulation of BTs

**Figure 1.**
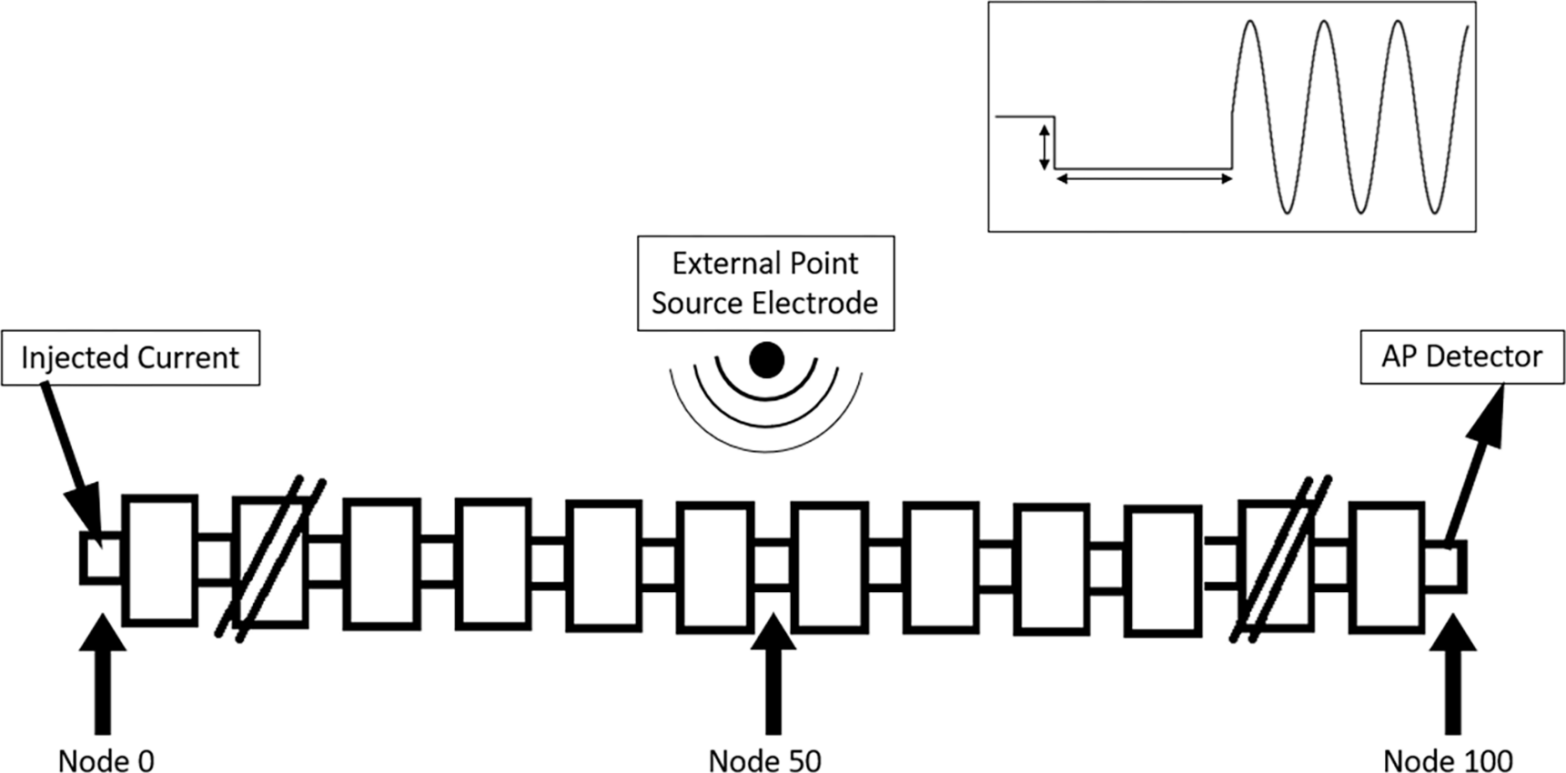
Simulation setup of a 101 node myelinated axon, with an injected current to generate test action potentials at node 0. The external blocking electrode is placed 1 mm from the center node (node 50) to inject the CROW. The waveform is shown in the inset and is composed of an initial cathodic DC followed by the KHFAC. Action potentials generated at node 0 that were not blocked by the external electrode were detected at node 100.

A 101 node MRG axon model was used with an external electrode modeled as a single point source 1 mm from the axon over the central node (figure 1) and placed in an infinite homogenous isotropic medium (resistivity = 500 Ω cm) [33]. For KHFAC block, a current-controlled sinusoidal KHFAC waveform (10 kHz) was generated at this electrode to produce a blocking stimulus. Ten kilohertz was chosen since it produces more onset in the model than 20 kHz, where the latter frequency was used for *in-vivo* experiments. For DC block, a cathodal current was generated. An internal electrode at one end of the axon (node 0) was used to inject test pulses, after the blocking current had been delivered for 40 ms. A successful block was defined as the condition where no action potential propagated to the node at the opposite end of the axon (node 100). The amplitude of the blocking waveform was varied in a binary search pattern (with a resolution of 1 *μ*A) to find the minimum current at which block occurred, termed the BT. This process was repeated for both DC and KHFAC waveforms. This procedure has previously been described [10].

#### 2.1.3. Simulation of KHFAC onset and reduction with a DC pulse

Within the model, onset is defined as the set of action potentials that propagate out from the center of the axon when the KHFAC is first initiated. To quantify the reduction in KHFAC onset response, we first identified the BTs for KHFAC (at 10 kHz) and cathodic DC waveforms using a 7.3 *μ*m diameter axon. Trials were simulated to have a DC pulse that varied in both amplitude and duration followed immediately by KHFAC at BT (figure 1 inset). DC durations ranged from 10 ms to 100 ms in increments of 10 ms, and DC amplitudes ranged from 0 to 3 times the DC BT in linear increments.

Every trial ran for 200 ms with an initial 10 ms of steady state. This allowed at least 90 ms of KHFAC delivery after the DC pulse to ensure the entire onset was observed. Throughout the 200 ms all action potentials that propagated from the center of the axon to the distal end were recorded with their arrival times. The DC pulse generated at most one action potential, so the shortest pulse duration of 10 ms was sufficient for making a quantifiable distinction between DC and KHFAC onsets. The DC was not ramped in these simulations although that could have been utilized to prevent generation of the single action potential at the beginning of the DC pulse.

#### 2.1.4. State space analysis

Additional simulations were conducted to compare the onsets from KHFAC with and without DC. These trials were conducted with a 10 *μ*m diameter axon. The external DC amplitude was set to twice the DC BT and the KHFAC was set to its own BT amplitude. During these matched trials, multiple variables were recorded at every time step of the simulation, specifically the *m* and *h* gating variables of all nodes. These two gates describe the behavior of the fast sodium channel which has been previously studied and determined to be of significance in KHFAC block [11]. These two gates can be plotted against each other for any node of interest to visualize the activation and inactivation levels of the fast sodium channel. Plots were generated to show the moving average of the two gate variables over each cycle of KHFAC. The average value during KHFAC approaches a constant, allowing analysis of the quasi steady-state to be a point instead of a cycle. The output of the moving average is a high dimensional set of oscillations and some of these can oscillate at high enough amplitude to become unstable and propagate out as an action potential.

### 2.2. *In-vivo* experimental protocols

#### 2.2.1. Animal preparation

**Figure 2.**
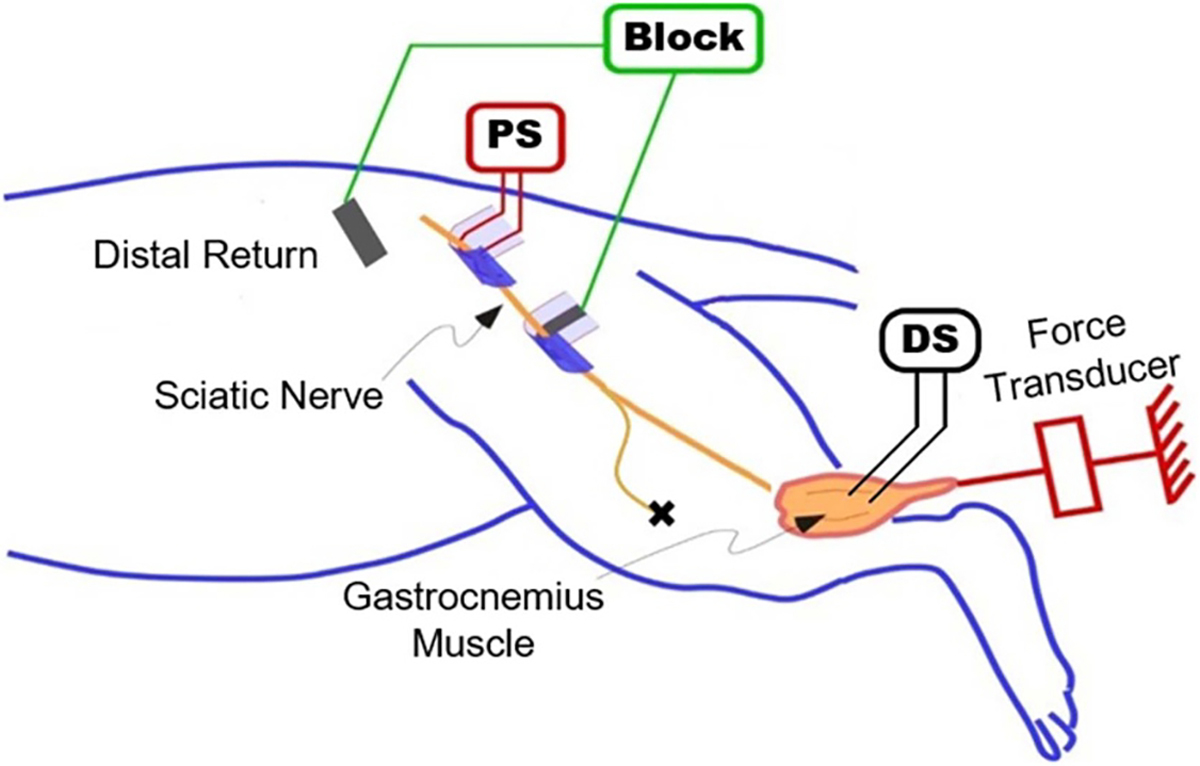
*In-vivo* experimental setup. A bipolar electrode was placed on the proximal portion of the sciatic nerve to elicit twitches from the gastrocnemius muscle. The block electrode was placed more distally on the sciatic nerve, with a return electrode placed just under the skin. The gastrocnemius tendon was attached to a force transducer to record muscle twitches. Stimulating needle electrodes were placed in the muscle to elicit distal stimulation.

Acute experiments were conducted with adult Sprague–Dawley rats. All procedures were approved by the Case Western Reserve University Institutional Animal Care and Use Committee prior to experiments and followed our previously published techniques [5]. Animals were induced and maintained under anesthesia with Isoflourane (1%–3%) over the course of the experiment, which typically lasted 5–6 h. The hindlimb was shaved, and an initial incision was made over the sciatic nerve near the iliac crest. The superior gluteal nerve was cut and a proximal stimulating (PS) bipolar cuff electrode was placed on the sciatic nerve. A second incision exposed the sciatic nerve near the bifurcation of the tibial and common peroneal nerves. The common peroneal nerve was cut, and a carbon coated monopolar electrode was placed on the tibial nerve (described in the next section). A return electrode was placed subcutaneously in the thigh. The Achilles tendon was exposed, cut distally and attached to a force transducer (Entran, Fairfield, NJ). Distal stimulation (DS) needle electrodes were placed directly in the gastrocnemius muscle. This setup is depicted in figure 2.

#### 2.2.2. Stimulation/recording

PS and DS were provided from a Grass stimulator (Grass Technologies, S88) passing through a Photoelectric Stimulus Isolation Unit to provide constant current output (Grass Technologies, PSIU6). The blocking waveform was designed in Matlab (Mathworks, R2018a). The blocking waveform was then generated from a Labview DAQ (USB-6363) at 500 kHz which sent a voltage signal to an isolated linear voltage to current stimulator (Caputron, C-LCI1107 H). The voltage across a 100 Ohm resistor was also monitored to accurately record the current being delivered to the nerve. Signals were amplified (Cambridge Electronic Design (CED) 1902) and digitized at 100 kHz (CED 1401).

#### 2.2.3. Carbon coated monopolar electrode for block

The blocking electrode was coated with a novel carbon ink [24]. The electrode was a J-cuff style electrode [34], with platinum as the base metal between two silicone sheets with an exposed window. The carbon ink consists of 3 g carbon black (YP-50 Kuraray, Canoga Park, CA) combined with 6 g of N-methyl pyrrolidone (NMP) and finally 3 g of 10% polyvinylidene fluoride diluted in NMP. A small amount (5–10 *μ*l) of the carbon ink was deposited on the platinum surface and baked at 200 °F for 20 min. The resulting electrode has very high capacitance [24]. The total charge capacity of each electrode was measured before each experiment via cyclic voltammetry using a Solartron Inc., Model 1280B Potentiostat with a sweep rate 10 mV s^−1^, voltage range of −0.255 to +1.20 V, and sampled at 10 Hz. The electrodes were placed in a bath of Ringers solution with a Ag/AgCl reference (BASi RE5B) and a large platinum return in a three-electrode measurement configuration. The *Q* value, or the total amount of charge that can be delivered, was calculated by integrating the current in the capacitive region of the electrode.

#### 2.2.4. Waveform

**Figure 3.**
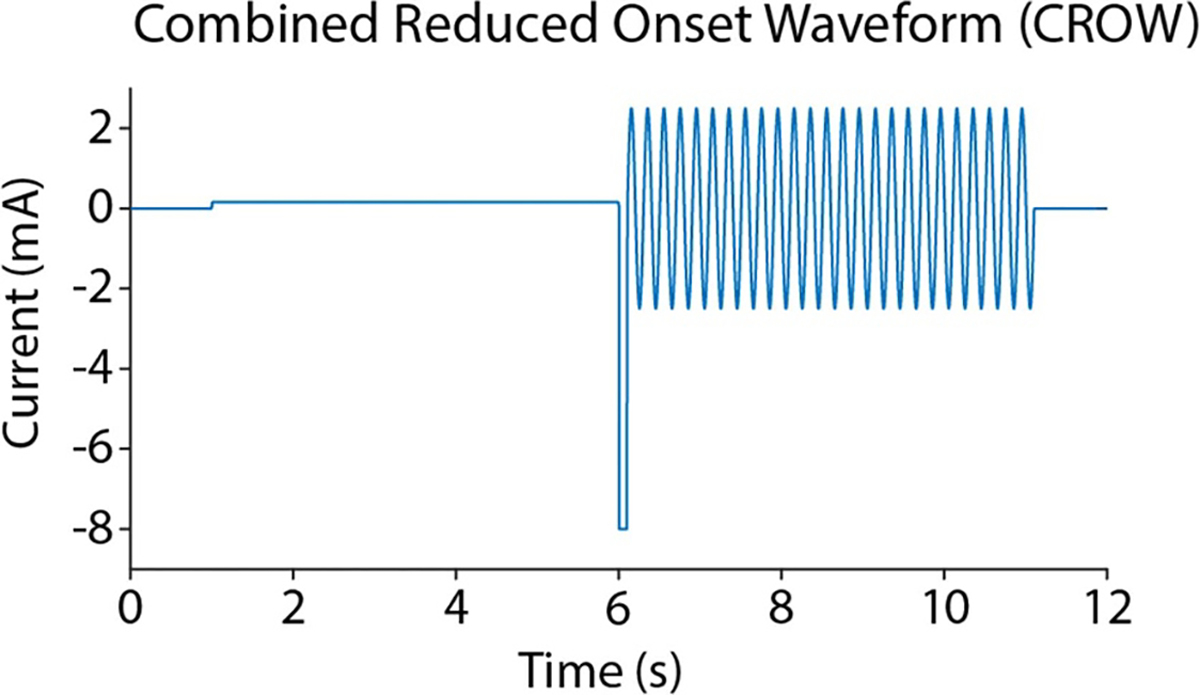
An example of the *in-vivo* combined waveform. The DC is delivered first, with the anodic phase leading the cathodic phase to balance the charge. KHFAC is initiated immediately after the cathodic pulse. In the second set of experiments, a time gap is inserted between the DC and the KHFAC (not shown in the figure).

Figure 3 depicts the waveform tested in the study. The first phase is a charge balanced DC pulse, consisting of a long anodic phase followed by a short cathodic pulse. The anodic phase was kept low (10% of cathodic amplitude) to avoid activation or block of the underlying neural tissue and served to balance the charge delivered during the cathodic phase [22]. The KHFAC began immediately after the DC phase, and was always a 20 kHz sine wave at the KHFAC BT [35]. In one group of experiments, a time gap was inserted between the DC and the KHFAC (not shown in the figure).

#### 2.2.5. Experimental protocol

Two protocols were followed to obtain the data in this work. In each protocol BTs were measured for both DC and KHFAC (described in the next paragraph). In the first protocol, the DC was followed immediately with the KHFAC. There were three randomized experimental blocks in which the amplitude (1–10 mA) and duration (100 ms–2 s) of the cathodic DC pulse were systematically varied (*N* = five animals). In each case the DC was followed by a 20 kHz KHFAC signal at the KHFAC BT. The DC amplitudes were based on the DC BT. Each trial captured the onset response for each amplitude/duration combination for a total of three repeats. The onset was quantified by its area, calculated as the integral under the force trace (force–time integral), and the maximum (peak) twitch force, which is the maximum force during the onset.

BTs were measured before each set of the three randomized sets in all protocols. For these trials, the PS was initiated before block. For KHFAC, the amplitude began at 10 mA peak to peak (PP) for 20 s [5]. This amplitude was then stepped down by 1 mA PP for 3 s at each value. The BT was defined as the lowest amplitude at which twitches were completely absent [5]. For DC, the cathodic amplitude began at 0.5 mA and was increased in 0.25 mA for 3 s until the twitches disappeared. The lowest value at which twitches completely disappeared was designated as the BT.

For the second protocol of experiments, a time gap was inserted between the end of the DC pulse and the beginning of the KHFAC waveform (*N* = six total animals with two preliminary). Based on the results from the first protocol, the DC pulse amplitude was set to minimize the onset, typically 6–8 times the DC BT for 100 ms. The time between these two phases was increased on a logarithmic scale in the range of 10 ms–20 s and the resulting onset and onset reduction was tabulated. Before inserting the gaps, the onsets due to KHFAC and DC were separately measured. Again, three randomized experimental sets were performed, resulting in three measurements per animal.

## 3. Results

### 3.1. Simulation results

**Figure 4.**
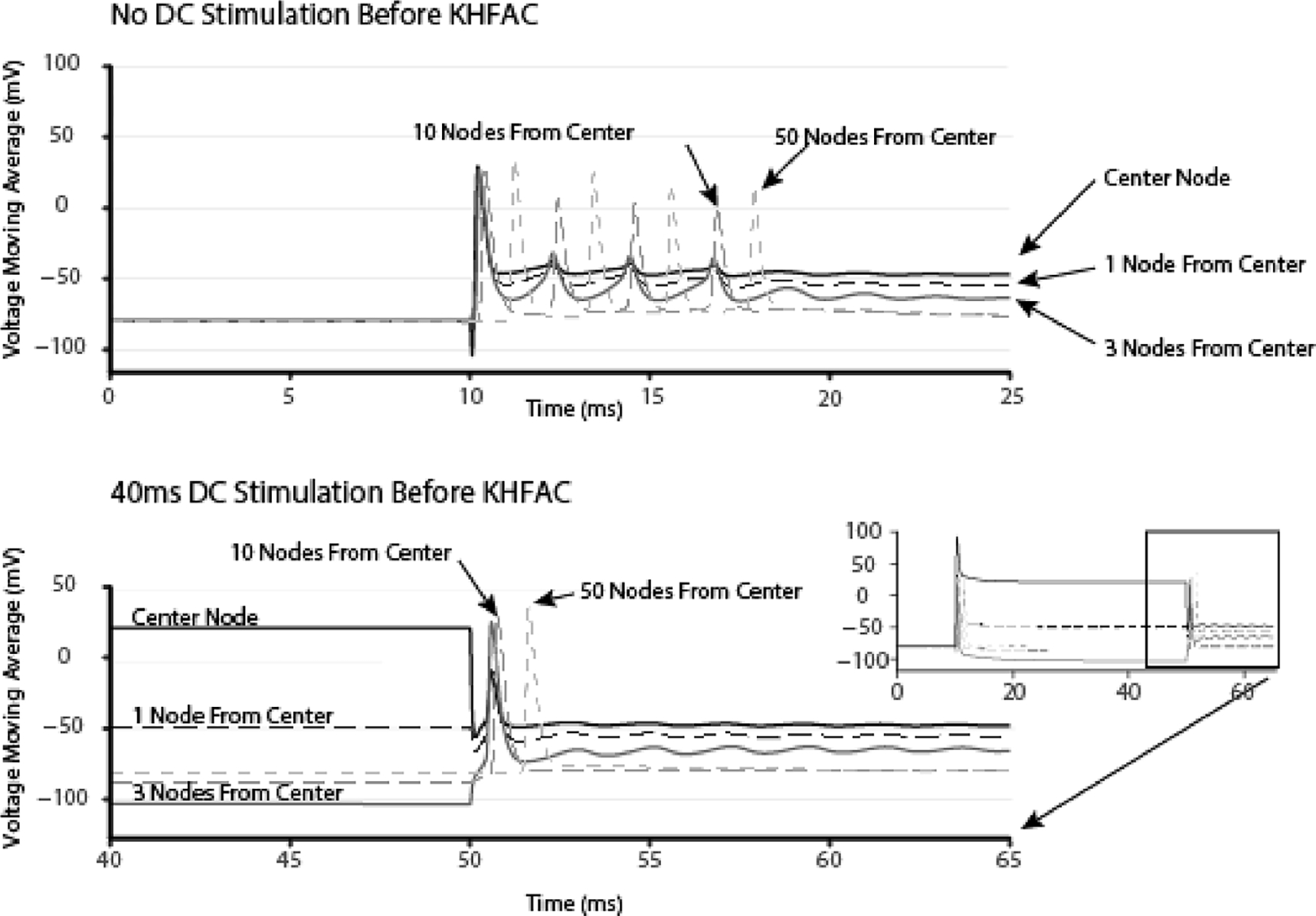
Simulation example of reduction in the KHFAC onset using a preceding DC cathodic pulse. Transmembrane voltages are shown as a moving average over an entire cycle of the 10 kHz sinusoidal current injection. The added DC shows up on the trace as a constant voltage preceding the KHFAC. Without DC, four conducted APs are generated when the KHFAC is applied. With DC there is only one conducted AP.

**Figure 5.**
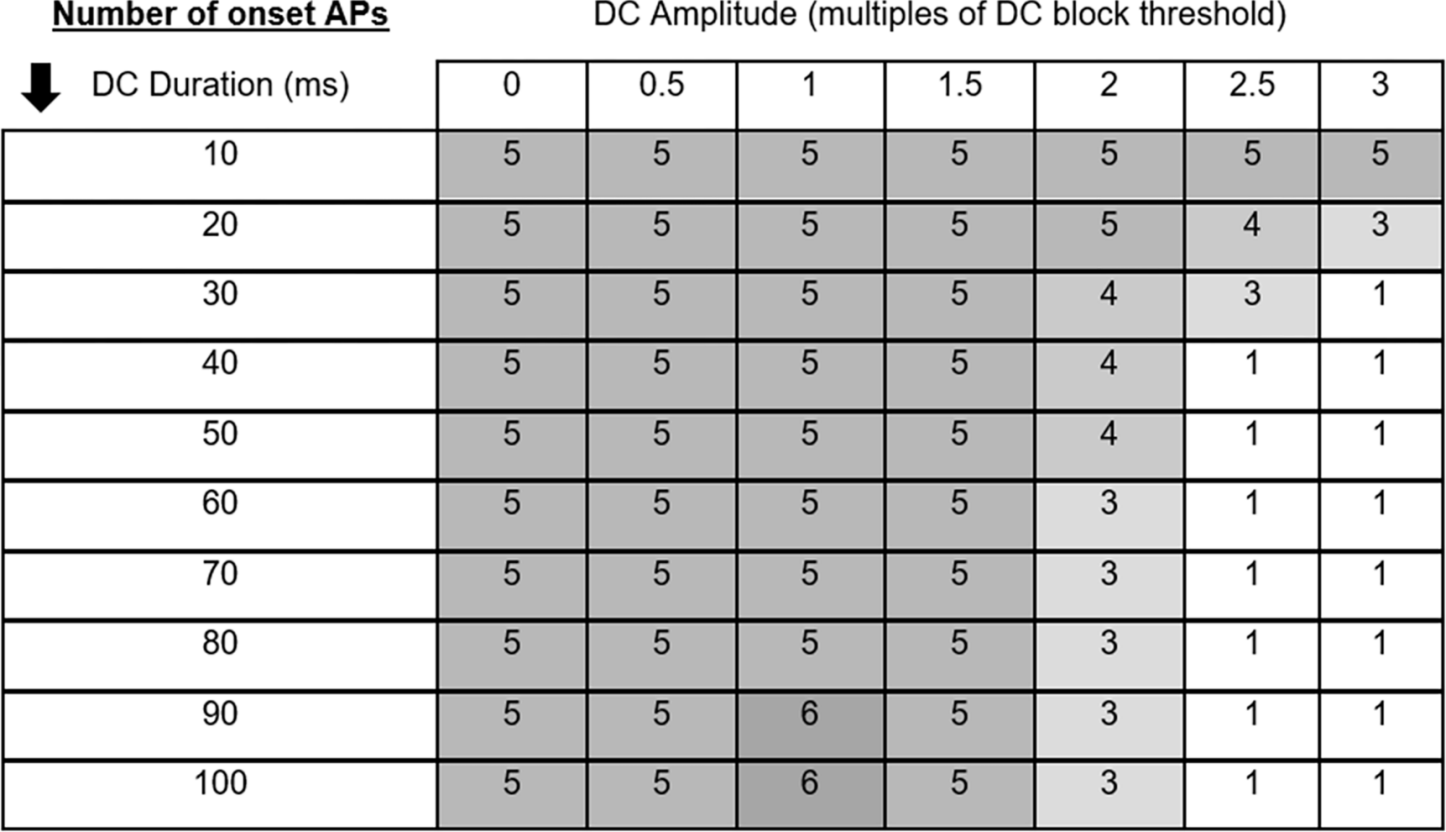
In-silico KHFAC onset (measured as the number of action potentials) when preceded by varying amplitudes and durations of DC pulses. Maximum reduction is obtained using a DC amplitude three times the DC block threshold with a duration of 30 ms.

DC BTs for the 7.3 *μ*m and 10 *μ*m axons were −0.269 mA and −0.305 mA, respectively (consistent with [4]). The 10 kHz KHFAC BT were 0.712 mA and 0.565 mA peak respectively (consistent with [32] and [10]). In the model, the KHFAC produces a short onset consisting of 3–5 action potentials. This onset is comparable to a Phase I onset, as described in the introduction. Figure 4 shows the averaged voltage of several nodes for a 10 *μ*m axon. The KHFAC generated four propagating action potentials when no DC pre pulse was utilized and only one onset action potential when the DC pre pulse was used. Figure 5 shows the results from a series of DC amplitude/durations on the number of action potentials in the onset response of the 7.3 *μ*m axon diameter. Increasing DC duration beyond 40 ms within the model has minimal to no additional benefits, but the DC amplitude has an effect on the reduction of the KHFAC onset. KHFAC alone at BT in this model produces an onset of five action potentials. This onset was reduced for all amplitudes over 1.5 times BT and durations over 20 ms. In general, a DC pulse of both sufficient amplitude and duration could reduce KHFAC onset in all fiber sizes tested.

**Figure 6.**
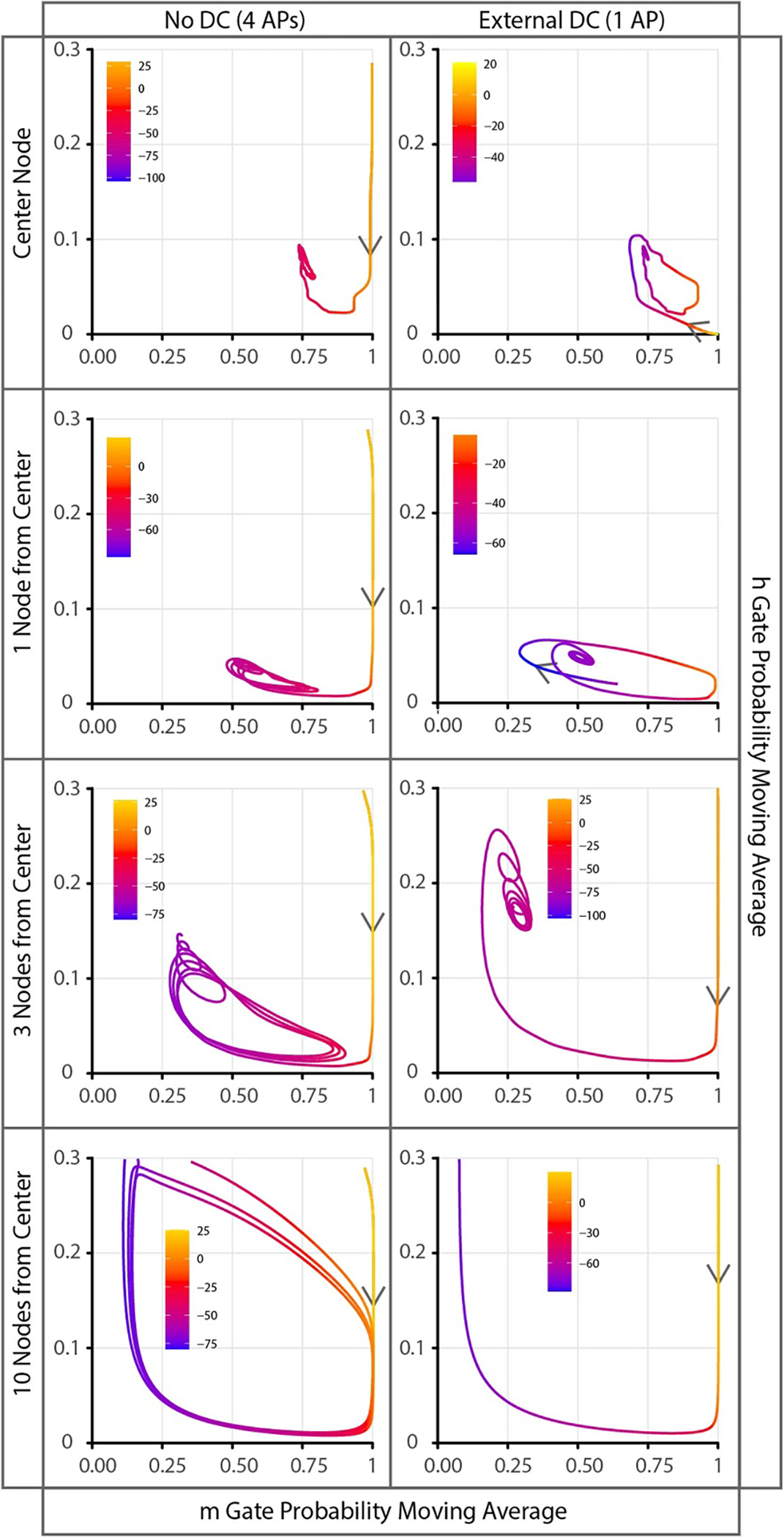
State space plots of the sodium channel gate probabilities (*x*-axis = *m* Gate, *y*-axis = *h* Gate) each showing individual nodes 50 (center), 49 (1 from center), 47 and 40. All values are a moving average over the entire cycle of KHFAC. Trajectories are color coded to show the instantaneous transmembrane voltage potential. The left column shows the sodium channel gates for 15 ms of KHFAC at BT with no DC stimulation which had four onset action potentials. The right column shows the same 15 ms time window but with a 40 ms DC pulse at 2x the DC BT immediately before the KHFAC which reduced the onset activity to a single action potential. The arrows denote the direction through time which leads toward the quasi-steady state. All plots with no DC pulse start from the initial rest state (*m* ≈ 0.073 and *h* ≈ 0.620). It should be noted that the y axis range is limited to 0–0.3 to better view the quasi steady-state. The initial state of KHFAC when preceded by a DC pulse is different for each node. This is seen by the different starting locations for each path. Depolarized nodes (center and one node from center) tend toward *m* = 1.000 and *h* = 0.000 and hyperpolarized nodes (three nodes from center) tend toward *m* = 0.000 and *h* = 1.000. These changed initial conditions of KHFAC allow for each node to reach the eventual steady state with fewer oscillations propagating out into action potentials. A video of this data is available in the supplemental material online.

State space analysis comparing no DC versus KHFAC with added DC showed KHFAC onsets consisting of 4 and 1 action potentials respectively (figure 6—see supplemental material for a video of this data (available online at stacks.iop.org/JNE/18/046010/mmedia)). Each action potential in the phase plane is seen as a clockwise path. The resting state of all nodes is initially at *m* ≈ 0.073 and *h* ≈ 0.620. Depolarization by the DC extracellular stimulation sets the gates of the central node (or surrounding nodes depending on the extent of depolarization) into a state with *m* ≈ 1.00 and *h* ≈ 0.00. This allows the transition from DC to KHFAC to have lower amplitude oscillations of gating variables as they approach the quasi steady-state (figure 6). Lower amplitude oscillations lead to fewer oscillations propagating out as conducted action potentials. It should be noted that the virtual anodes created during external cathodal DC stimulation have the opposite effect [4]. Nodes within the virtual anode region are hyperpolarized, and require a larger change in the gating variable’s state to reach their quasi steady-state (causing a single initial action potential in every trial shown).

### 3.2. Experimental results

#### 3.2.1. DC pulse amplitude/duration

**Figure 7.**
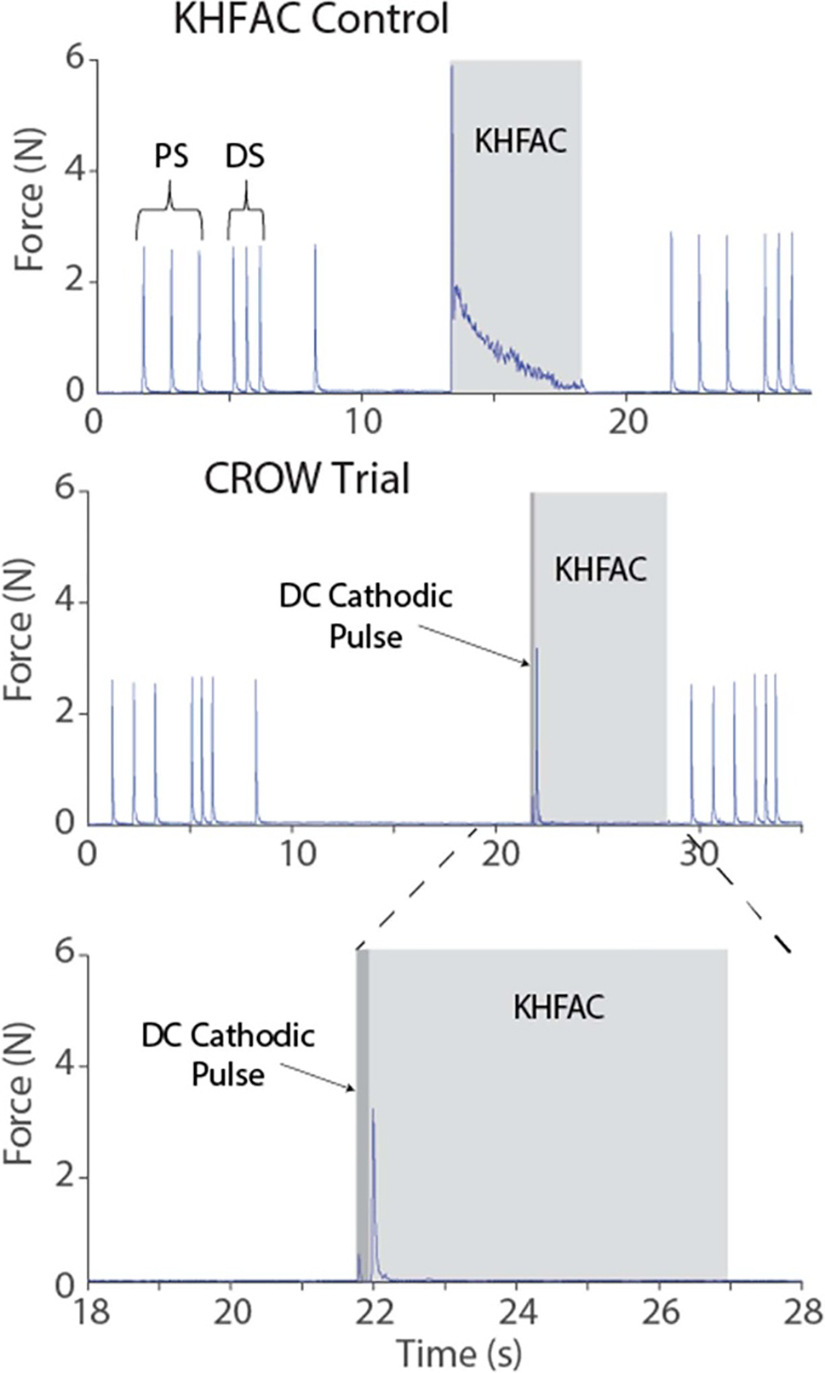
*In-vivo* example trials. The top panel shows a pure KHFAC trial which serves as a control. The second panel shows a CROW trial. The third panel is a zoomed in portion of the CROW trial to show the onset in more detail. The triplets of twitches before and after each trial represent the proximal and distal stimulation, respectively. The single twitch at ~8 s is generated by turning on the blocking stimulator (artifact). The light gray shows when the KHFAC is on in all three panels, while the darker gray areas/arrows on the middle and lower panels shows when the DC cathodic pulse is applied. The DC pulse causes a small twitch before the onset twitch (clearly seen in third panel), but the onset dissipates in ≪1 s as compared to >5 s in the control.

**Figure 8.**
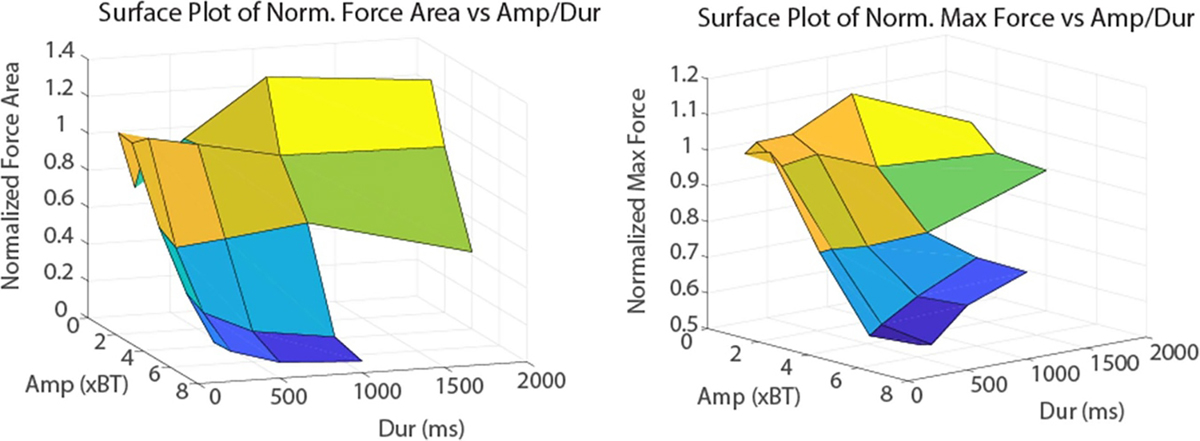
Surface plots of *in-vivo* onset reduction. The left panel shows a surface plot of the onset area, normalized to the onset due to KHFAC alone. The *x*-axis shows the amplitude of the DC as a multiple of the block threshold (BT) and the *y*-axis shows the duration in milliseconds. The plot demonstrates that little onset reduction is achieved when the amplitude is 1—2*x* the BT, but is significantly reduced with 4—8*x* the BT. The duration of pulse has a lesser effect but decreases the onset with increasing pulse duration. The right panel shows the reduction in the maximum force, which follows a similar trend but with less reduction (note differences in scale).

Complete block was achieved in all animals. The BTs were 0.88 ± 0.37 mA for DC and 4.82 ± 1.01 mA PP for KHFAC. The charge capacities for the electrodes used in both sets of animals was 42 ± 15 mC. Impedance, measured only in one animal, was 806 ± 7 Ω at 20 kHz. Figure 7 shows an example of a KHFAC onset response and the combined waveform onset response. The onset response due to KHFAC alone had decreased but not ended in the measured five seconds (top panel), while the onset due to the combined waveform lasted less than 300 ms (bottom panel). Figure 8, left panel, shows the average reduction in onset from the first set of animals tested (*N* = 5). The 3D surface plot shows the duration and amplitude of the DC pulse, while the *z*-axis shows the onset normalized to the control KHFAC trial. A one-way repeated measures ANOVA was conducted on the data. The average normalized area was averaged for the three repeats in each animal, and results were pooled by amplitude as a function of BT. ANOVA showed significant differences in normalized area across relative amplitudes (*p* < 0.01). DC pulses with 1–2 × BT did not significantly decrease the onset response (*p* > 0.05, *t*-test). DC pulses using 4 × BT (and all higher amplitudes) significantly decreased the onset response (*p* < 0.01, *t*-test). DC pulses of 6–8 times the DC BT showed the best onset reduction, with longer durations further increasing this reduction. The 2 s duration was not tested for 6–8 × BT to avoid exceeding the *Q* value. At the highest amplitude/durationtested (8×BT/1 s),onsetwasreduced by 94 ± 2%. Figure 8, right panel, shows the average reduction in the maximum (peak) twitch force of the onset, which also decreased to approximately 60% of the KHFAC onset response maximum force. Regression analysis (not shown) of the onset reduction versus the control onset showed a linear correlation, such that the onset reduction was proportional to the control onset (*t*-test of the slope, *p* < 0.05).

#### 3.2.2. Onset contributions

**Figure 9.**
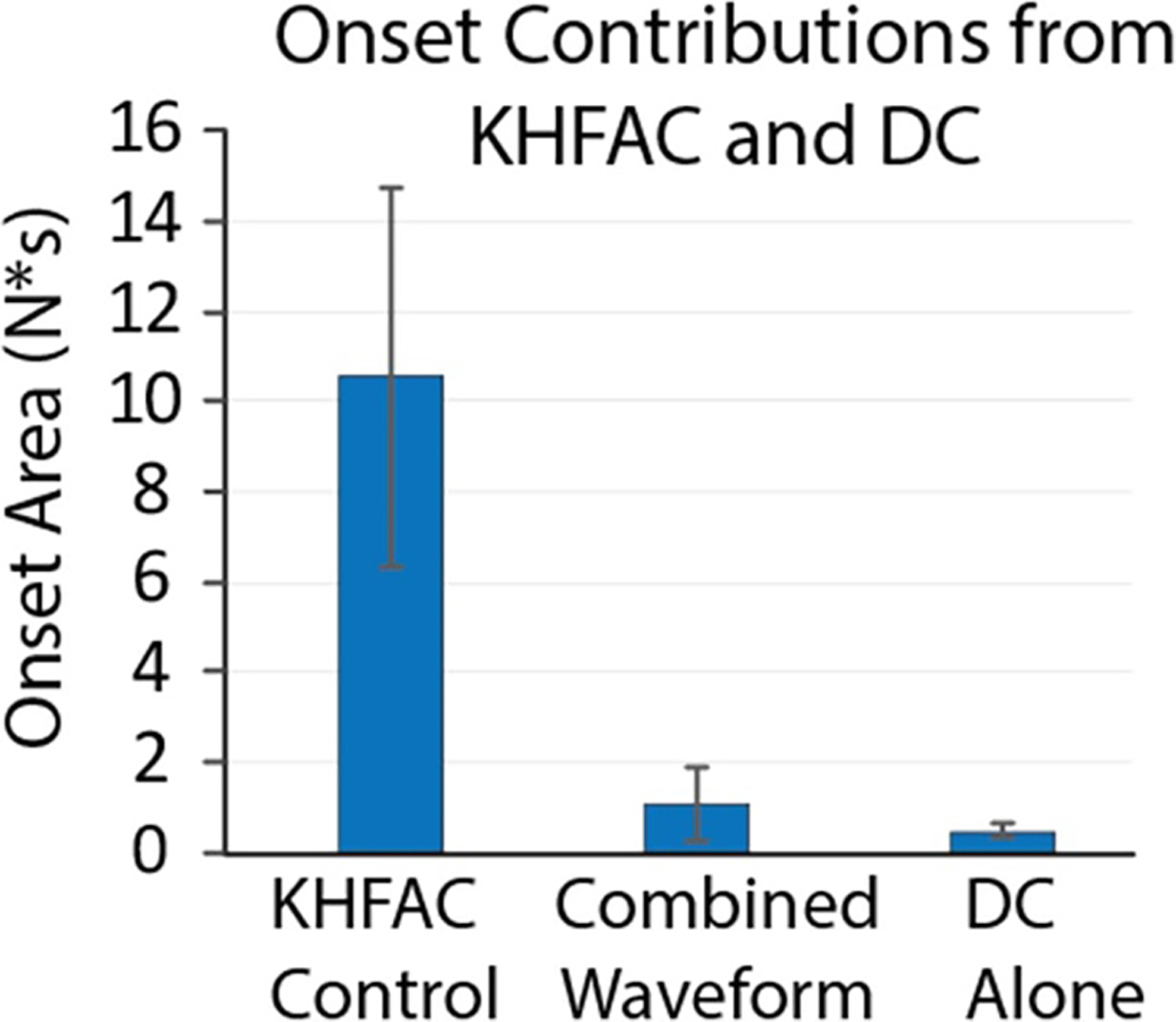
Onset contributions. The amount of onset due to KHFAC alone, DC alone, and the CROW are shown. The CROW had >90% less onset than the KHFAC control, and nearly half of that residual onset could be attributed to the DC portion of the waveform. DC injected with a short amplitude ramp would eliminate this portion of the onset produced by the DC pulse.

Although onset can be eliminated from DC block using a ramp, the CROW is implemented using a DC rectangular wave. This DC also produces onset without mitigation using an amplitude ramp. Therefore, the total onset mitigation of the CROW is in reality the sum of the KHFAC onset and the DC onset; onset area was calculated during KHFAC delivery, although the DC twitch force also appears during this period due to conduction delays. To determine the relative contribution of each, the onset due to KHFAC alone, the DC pulse alone, and the CROW were measured before each of the three experimental repeats in the second set of animals. These results are tabulated in figure 9. KHFAC was again delivered at BT, and DC was delivered at 6 × BT for 100–200 ms. KHFAC and DC BTs were measured before each experimental repeat. Similar to the first set of experiments, the combined waveform reduced the onset due to KHFAC by >85%. The onset from the DC pulse alone accounted for roughly half of this reduced residual onset.

#### 3.2.3. Delayed recovery from DC pulse

**Figure 10.**
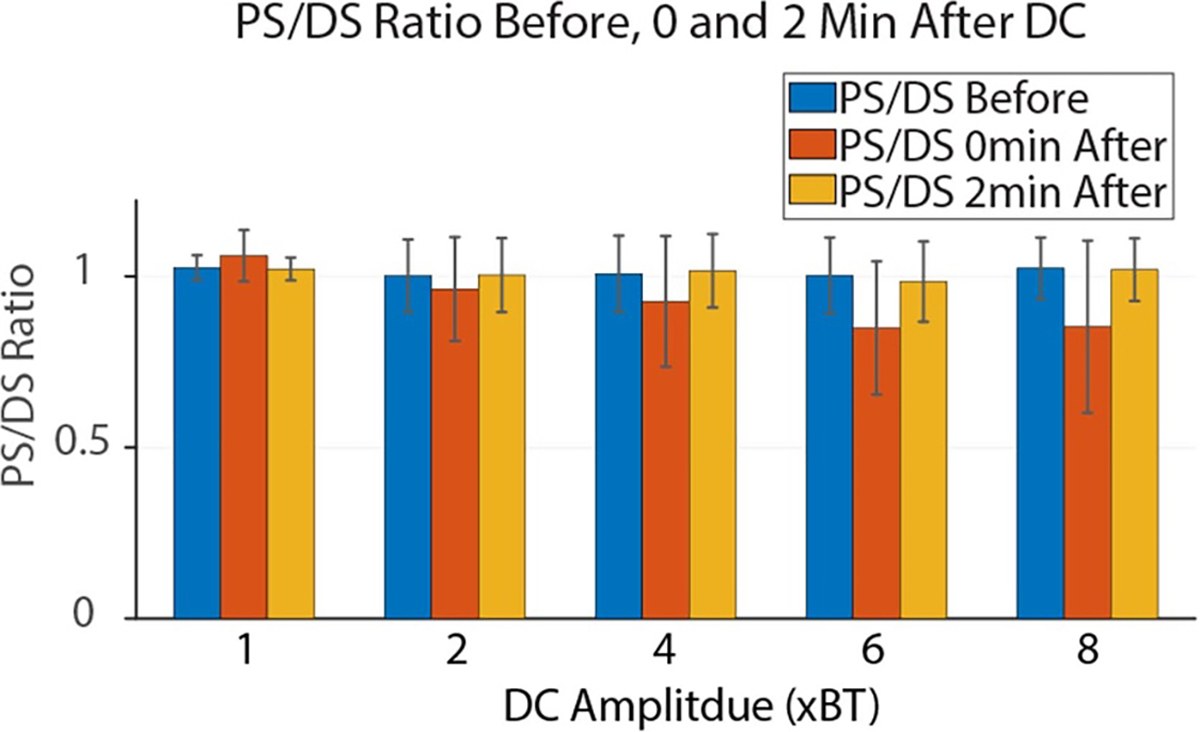
Ratio of the twitch heights elicited by proximal and distal stimulation. Each bar represents the mean and standard deviation of the ratio before, immediately after and 2 min after every trial for a given DC pulse amplitude in each of the animals tested. The data show that the PS/DS ratio was reduced after DC pulses with higher amplitudes. However, the ratios return to 1 by the beginning of the next trial, indicating that the reduction in proximal stimulation was temporary, fully recovering within the 2 min rest period.

Figure 10 shows the ratio of the PS and the DS across all trials in the first set of animals. This ratio is used as a measure of delayed recovery of conduction or nerve damage; a PS/DS ratio of less than one suggests localized delayed recovery or nerve damage, while a ratio of one represents normal conduction. The PS/DS ratio was calculated within each trial, before and after the CROW. A repeated measures ANOVA was again conducted, which showed significant differences between groups (amplitude) (*p* < 0.01). This ratio was below one for DC pulses at or above 4 × BT immediately after the CROW within each trial (*p* < 0.05, one sided *t*-test). However, when the PS/DS ratio at the beginning of each trial was compared to that at the beginning of the next trial, it was never significantly below one for any set of trials before using the combined waveform (*p* > 0.05, one sided *t*-test). This means that the PS/DS decrease was temporary and recovered to normal by the start of the next trial. The next trial started 2 min after the end of the previous trial. Linear regression analysis showed that the absolute amplitude of the pulse was more predictive of PD/DS ratio as compared to either relative amplitude or total charge. Over the course of the experiment, the absolute value of the twitch height declined, specifically in the third set. This decrease was approximately 10% from the first set, and is likely due to fatigue and general decline from anesthesia. The PS/DS ratio though held at one throughout the experiments.

#### 3.2.4. Gap effects

**Figure 11.**
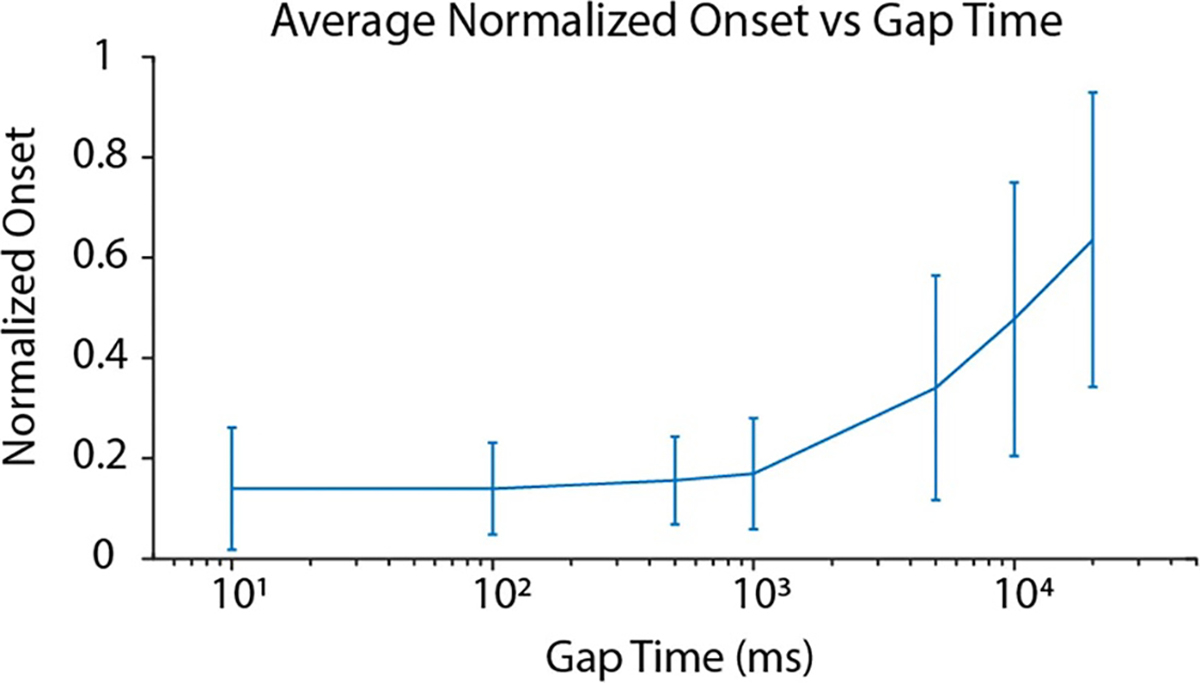
Effect of gap time. The *x*-axis shows the time between the DC and KHFAC while the *y*-axis shows the resulting onset area, normalized to the control KHFAC. The line shows the mean and standard deviation for all trials for all the animals tested. The gap times began with 10 ms and increased logarithmically to 20 s. The onset does not begin to increase (as compared to the control KHFAC) until a gap of one second is introduced, and continues to increase until 20 s, the longest gap time measured in this study.

Figure 11 shows the effect of introducing a gap between the cathodic DC and the KHFAC in the combined waveform on the onset response using the same amplitude/duration as the onset contribution trials. The reduction in onset is similar until nearly 1 s was introduced between the DC and KHFAC portions of the waveform, implying that the DC affected onset reduction for that duration. Beyond that duration of 1 s, the onset increased with increasing gap times, towards the level of onset without the CROW, approaching but not quite reaching 1 (full onset) even after 20 s, which was the longest time tested. This suggests that the DC pulse has an effect over a long duration on onset reduction.

#### 3.2.5. Ramped DC waveform

**Figure 12.**
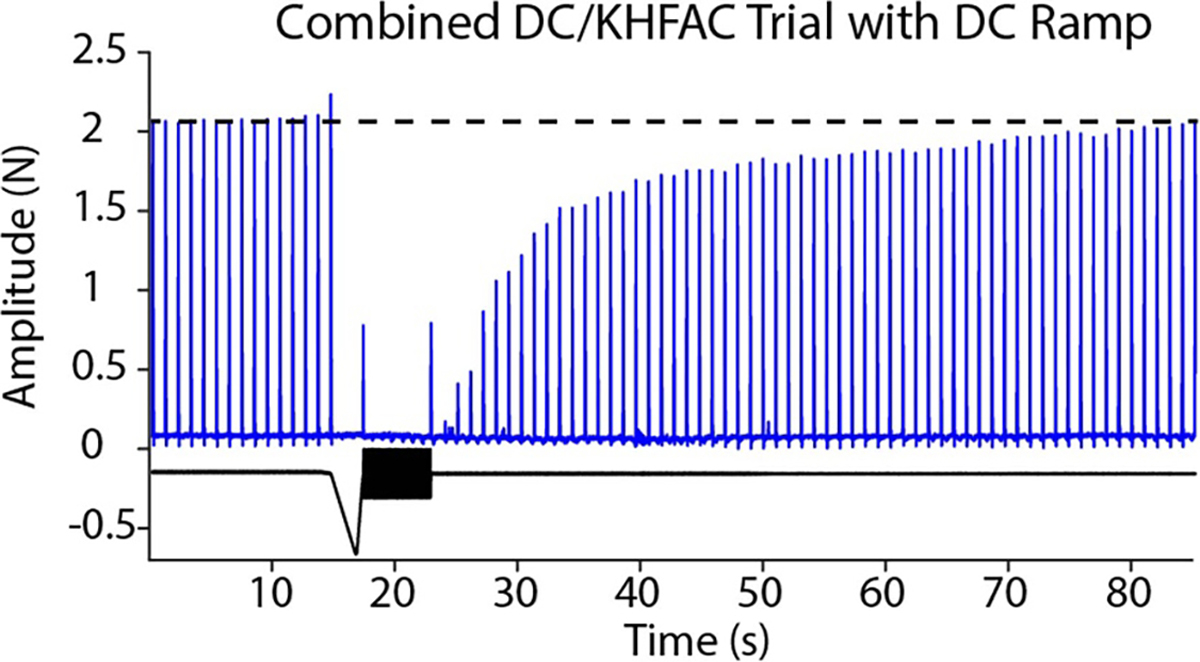
Ramped DC waveform. During the above trial, the DC was ramped down to the plateau value before ramping back up in 500 ms. The ramp helps eliminate the neural activation due to DC, leaving only a small onset twitch associated with the KHFAC. Also seen are an ‘off’ response when the KHFAC is terminated and the slow recovery of the proximal twitches, which are provided at 1 Hz throughout the trial. Full recovery occurs in ~60 s from the end of the CROW.

In a few trials, the DC waveform was ramped to eliminate the onset from the DC figure 12 shows a single trial in which the DC was ramped down to the necessary cathodic DC amplitude, and then quickly ramped back to zero within 500 ms before the KHFAC was initiated. Ramps can reduce or eliminate the portion of the onset due to DC (shown in figure 9). Proximal stimulation is provided throughout the trial at 1 Hz and shows that the nerve is blocked once the DC is initiated, and demonstrates the delayed recovery which in this trial takes ~60 s to fully recover. This trial also shows an ‘off’ response when the KHFAC is terminated; the cause of this response is not fully understood, but could be due to charge buildup during the current controlled KHFAC.

## 4. Discussion

In this work the effect of using a DC pulse before initiating KHFAC was investigated through computer simulations and *in-vivo* experiments. A DC pulse which is higher in amplitude compared to the DC BT is necessary to significantly reduce the onset response, on the order of 6–8 times the DC BT for a short duration pulse (100–200 ms). The highest DC amplitude tested in this work was 10 mA, due to equipment limitations; even higher DC amplitudes might further improve this technique. The CROW reduced the onset force-time integral largely by reducing the duration of the onset. It also reduced the resulting peak or maximum generated onset force, albeit to a lesser extent. Regression analysis (not shown) suggests that combining this waveform with other techniques which reduce the onset response, such as improved electrode design, could maximize the utility of the proposed waveform. This work shares many similarities to another onset reduction technique utilizing DC and KHFAC [27]. In this prior work, a KHFAC electrode was implanted with two flanking DC electrodes. The DC electrodes were activated before initiating KHFAC, thereby blocking the onset before it reached the end-organ. This work suffered many drawbacks, however, including the need for three separate electrodes and the associated implant sites/hardware, etc. This technique could also not guarantee no/minimal onset, as the DC electrodes are only capable of blocking for <10 s, and the onset could exceed this time frame. The present work operates in a different manner, preventing the onset from ever occurring using a single monopolar electrode and implant location.

### 4.1. Mechanism of action

The mechanism of action of the combined waveform is not fully understood, due in large part to the fact that the nature of the onset response is also not well understood and debated in the literature [10, 11, 36]. Our initial hypothesis in this work relied on having the DC block the onset response when the KHFAC was initiated, as discussed in [29]. However, through this work we found that the DC does not have to overlap the KHFAC at all in order to reduce the onset. Our hypothesis is that the DC pulse pre-conditions the ion channels, forcing them to the steady state values at which they oscillate around during KHFAC. This ‘preconditioning’ step would decrease the time for KHFAC to reach steady state, thereby reducing the onset. The simulation work supports this hypothesis, particularly the state space analysis. However, the effect of the gap times observed in figure 11 cannot be directly explained by this hypothesis. Modeling results show that the effect of the DC pulse should disappear within a few ms, as the ion channels rapidly return to their resting state in the absence of any DC. The gap data however suggests that some effect of the DC persists for seconds, and possibly for up to 60 s in some cases, which cannot be explained with the proposed mechanism alone.

### 4.2. Delayed recovery

Delayed recovery is seen in these experiments, as demonstrated in figures 10 and 12. This delay is likely due to a carry-over block effect, possibly from localized ionic accumulation [37]. Given the complete recovery of nerve activity in all cases, there was no evidence of nerve damage in any experiment, even after DC delivered at 8 × BT (max 10 mA). Temporal effects of DC block have been observed in other studies, particularly those involving separated nerve interface electrode or similar devices [24, 38]. With these devices, delayed block as well as delayed recovery has been reported. These studies have suggested that heating could contribute to these temporal effects, although that is not likely related to this study as the DC pulses are far too short to locally increase the temperature. Generating harmful electrochemical byproducts is also unlikely in this study, as the carbon-coated electrodes are capable of delivering ~10× the charge used in these experiments. Regression analysis showed that the absolute amplitude of the DC related to delayed recovery more strongly than the overall charge, which suggests that the amplitude alone may cause this effect. Electroporation could account for this phenomenon, since the absolute amplitude and not the duration are important for this effect, and the effect can take tens of seconds to recover [39]; however, this remains purely speculative at this point. This phenomenon remains an active area of research in our lab.

### 4.3. Ramped waveforms

In most trials, the cathodic DC presented as a pulse of varying amplitude and duration. As shown in figure 7, this DC pulse causes neural activation by itself, both at initiation of the DC as well as an off response when the DC ends. This activation can be alleviated by ramping the DC. The DC can be ramped as slowly as necessary to avoid activation on the leading edge. At the end of the DC, the ramp should occur within 500 ms to maximize the reduction in onset as evidenced by the gap experiments. Figure 12 shows an example of such a waveform, which creates a single twitch which is significantly smaller than a single proximally elicited twitch. While this waveform further decreases onset, the charge delivered is significantly higher for a ramped versus pulsed DC; specifically, this waveform required 15 mC of charge, while the DC pulses were generally ⩽ 1 mC to achieve onset reduction. This tradeoff will become important for clinical translation, as the maximum allowable charge for a given clinical electrode will limit the amount of DC that can be delivered.

### 4.4. Limitations

This work has several limitations. First, these experiments were performed under acute conditions, and therefore long term safety and efficacy for this waveform are not yet known. The electrodes used in this work are J-cuff style [34], which may not translate to chronic applications which typically require an electrode that fully encircles the nerve. The carbon coating utilized in these experiments has not yet been characterized for chronic efficacy. A clinically viable electrode which can deliver several millicoulombs of DC safely is crucial to advancing this technology.

## 5. Conclusions

In this work we have explored combining DC and KHFAC in the form of the CROW to reduce the onset response due to KHFAC alone. Short DC pulses injected just before initiating KHFAC were shown to greatly reduce the KHFAC onset response, in some cases by >95%. The mechanism of this waveform for onset reduction, particularly in light of the results of the gap experiments, is not well understood and need further investigation. Future work will include more detailed modeling and experimental studies to elucidate the mechanism and to test other electrode configurations and chronic safety.

## Supplementary Material

Eggers Supplement doc

Eggers video 1

Eggers video 2

## Figures and Tables

**Table 1.** Parameters used for MRG simulation of peripheral nerve axons.

Parameters	Values

Node length	1 *μ*m
Myelin attachment paranode length	3 *μ*m
Main paranode length	Diameter dependent
Internodal section length (× 6)	Diameter dependent
DC capacitance (C_-DC-_)^A^	2 *μ*m cm^−2^
Infinte frequency capacitance (C_∞_)^A^	1.1 *μ*m cm^−2^
Myelin capacitance	0.1 *μ*m cm^−2^
Axoplasmic resistivity	70 Ω cm
Periaxonal resistivity	70 Ω cm
Myelin conductance	0.001 S cm^−2^
Myelin attachment paranode conductance	0.001 S cm^−2^
Main segment paranode conductance	0.0001 S cm^−2^
Internode segment conductance	0.0001 S cm^−2^
Maximum fast Na^+^ conductance	3 S cm^−2^
Maximum persistent Na^+^ conductance	0.01 S cm^−2^
Maximum slow K^+^ conductance	0.08 S cm^−2^
Nodal leakage conductance	0.007 S cm^−2^
Na^+^ Nernst potential	50 mV
K^+^ Nernst potential	−90 mV
Leakage reversal potential	−90 mV
Resting potential	−80 mV
Temperature	37 °C

A Parameters of the frequency-dependent membrane capacitance (Howell *et al* 2015)

## Data Availability

The data that support the findings of this study are available upon reasonable request from the authors.
