# Supplementary material for: Combining direct current and kilohertz frequency alternating current to mitigate onset activity during electrical nerve block
Source: J Neural Eng. Author manuscript; Available in PMC 2022 Sep 26. (PMC9511888; doi:10.1088/1741-2552/abebed)
Supplement: Eggers Supplement doc [file NIHMS1830172-supplement-Eggers_Supplement_doc.docx]

Caption for supplemental videos:

**Supplemental material: State-space videos.** The two videos show the state-space plots over time. This data is the same as shown in Figure 6 of the text. The panels on the left represent standard KHFAC, while the right panels show the CROW. The first video is slowed to show the first several milliseconds. Note the different starting locations for each node. The second video shows the full onset response, with four action potentials in the KHFAC panel and only 1 in the CROW. Figure legend: Colors represent different nodes. Orange - center; red - 1 node from center; purple - 2 nodes from center; blue - 3 nodes from center; green - 4 nodes from center
